# Supplementary material for: Patient-derived models recapitulate heterogeneity of molecular signatures and drug response in pediatric high-grade glioma
Source: Nat Commun. 2021 Jul 2;12:4089. doi: 10.1038/s41467-021-24168-8 (PMC8253809; doi:10.1038/s41467-021-24168-8)
Supplement: Supplementary file 3 — Description of Additional Supplementary Files [file 41467_2021_24168_MOESM3_ESM.docx]

Description of Additional Supplementary Files

File Name: Supplementary Data 1

Description: Cohort of patient tumors and derivative PDOX and cell line models.

1a. Summary of clinical information, histopathology, DNA methylation classification and signature genes mutated for patient tumors, PDOXs, and cell lines, time to PDOX engraftment and short tandem repeat (STR) fingerprinting profile for model identification.

1b. Scores for methylation-based classification

File Name: Supplementary Data 2

Description: Sequence alterations in patient, PDOX and cell line cohorts

2a. List of signature genes and associated pathways recurrently mutated in pHGG

2b. Non-silent mutations in HGG signature genes identified in 57 samples across 21 lines (relates to Fig. 4)

2c. Non-silent somatic mutations in non-hypermutator patient tumors, PDOXs and cell lines with matched germline available

2d. Potentially pathogenic non-silent SNVs and INDELs in patient tumors, PDOXs and cell lines without paired germline available

File Name: Supplementary Data 3

Description: GSEA of genes differentially expressed in PDOX compared with patient tumors

3a. Genes down-regulated in PDOX samples compared to matched patient tumors (logFC < -1 & adj. P < 0.05)

3b. Significantly enriched (adj. P < 0.05) MSigDB Hallmark gene sets for the down-regulated genes in PDOX samples

3c. Genes up-regulated in PDOX samples compared to matched patient tumors (logFC > 1 & adj. P < 0.05)

3d. Significantly enriched (adj. P < 0.05) MSigDB Hallmark gene sets for the up-regulated genes in PDOX samples

File Name: Supplementary Data 4

Description: Results of high-throughput screening

4a. Column definitions for DR fits for 2D vs. 3D culture condition comparison

4b. DR fits for 2D vs. 3D culture condition comparison

4c. FDA single-point screen in 9 pHGG cell line models and human embryonic stem cell-derived neural stem cells (HNSC)

4d. Column definitions for the pHGG DR screens

4e. DR fits for 246 compounds profiled in 4 exemplar pHGG cell line models

4f. DR fits for 93 compounds profiled in 14 pHGG cell line models and 2 control cell lines (iAstro and HABS)

4g. Column definitions for Combinations Studies – Raw Data

4h. Combinations Studies – Raw Data

4i. Column definitions for Combinations Studies – Fits

4j. Combination Studies - Fits

File Name: Supplementary Data 5

Description: Pharmacokinetic analysis of paxalisib and mirdametinib

5a. Drug-drug interaction PK study design

5b. Non-linear mixed effect (NLME) plasma PK model parameter estimates for mirdametinib, 14 mg/kg PO, QD

5c. NLME plasma PK model parameter estimates for paxalisib, 8 and 10 mg/kg PO QD
