# Supplementary material for: Patient-derived models recapitulate heterogeneity of molecular signatures and drug response in pediatric high-grade glioma
Source: Nat Commun. 2021 Jul 2;12:4089. doi: 10.1038/s41467-021-24168-8 (PMC8253809; doi:10.1038/s41467-021-24168-8)
Supplement: Supplementary file 9 — Reporting Summary [file 41467_2021_24168_MOESM9_ESM.pdf]

nature research

Corresponding author(s): Suzanne J. Baker, Anang A. Shelat, Christopher L. TInkle and Jason Chiang

Last updated by author(s): Apr 11, 2021

Reporting Summary

Nature Research wishes to improve the reproducibility of the work that we publish. This form provides structure for consistency and transparency in reporting. For further information on Nature Research policies, see our Editorial Policies and the Editorial Policy Checklist.

Statistics

For all statistical analyses, confirm that the following items are present in the figure legend, table legend, main text, or Methods section.

n/a	Confirmed	
☐	☒	The exact sample size (*n*) for each experimental group/condition, given as a discrete number and unit of measurement
☒	☐	A statement on whether measurements were taken from distinct samples or whether the same sample was measured repeatedly
☐	☒	The statistical test(s) used AND whether they are one- or two-sided<br>*Only common tests should be described solely by name; describe more complex techniques in the Methods section.*
☒	☐	A description of all covariates tested
☐	☒	A description of any assumptions or corrections, such as tests of normality and adjustment for multiple comparisons
☐	☒	A full description of the statistical parameters including central tendency (e.g. means) or other basic estimates (e.g. regression coefficient) AND variation (e.g. standard deviation) or associated estimates of uncertainty (e.g. confidence intervals)
☐	☒	For null hypothesis testing, the test statistic (e.g. *F*, *t*, *r*) with confidence intervals, effect sizes, degrees of freedom and *P* value noted<br>*Give P values as exact values whenever suitable.*
☒	☐	For Bayesian analysis, information on the choice of priors and Markov chain Monte Carlo settings
☐	☒	For hierarchical and complex designs, identification of the appropriate level for tests and full reporting of outcomes
☐	☒	Estimates of effect sizes (e.g. Cohen's *d*, Pearson's *r*), indicating how they were calculated

Our web collection on statistics for biologists contains articles on many of the points above.

Software and code

Policy information about availability of computer code

Data collection	Standard Illumina instrumentation software (HiSeq2000 for most samples) and CGI software were used to collect the DNA and RNA sequencing data reported in this study.
Data analysis	DNA methylation array data analysis with t-SNE algorithm (Rtsne package v.0.11), Copy-number alteration (CNA) analysis was done using the conumee package (v 1.18.0) with default parameters, We used BWA (v 0.5.9), STAR (v 2.3.0), XenoCP (v 3.0.0), Bambino (v 1.6), CONSERTING (v 1.0), IGV (v 2.7.2), ProteinPaint web server (proteinpaint.stjude.org), CICERO (v 0.3.0), HTSeq-Count (v 0.11.2), edgeR (v 3.28.0) and limma (v 3.42.0) for the WGS, WES, and RNA-seq data analyses. Tools are published and publicly available as cited in Methods.

For manuscripts utilizing custom algorithms or software that are central to the research but not yet described in published literature, software must be made available to editors and reviewers. We strongly encourage code deposition in a community repository (e.g. GitHub). See the Nature Research guidelines for submitting code & software for further information.

Data

Policy information about availability of data

All manuscripts must include a data availability statement. This statement should provide the following information, where applicable:

- Accession codes, unique identifiers, or web links for publicly available datasets
- A list of figures that have associated raw data
- A description of any restrictions on data availability

DNA methylation profiles profiling data generated in this study are available in the Gene Expression Omnibus (GEO) database, accession GSE152035 [https://www.ncbi.nlm.nih.gov/geo/query/acc.cgi?acc=GSE152035]. Next-gen sequencing data generated in this study have been deposited at the European Genome-Phenome Archive (EGA), which is hosted by the European Bioinformatics Institute (EBI), and are available under the indicated accession numbers for whole-genome (EGAS00001005159) [https://ega-archive.org/search-results.php?query=EGAS00001005159] , whole-exome(EGAS00001005160) [https://ega-archive.org/search-

results.php?query=EGAS00001005160] and RNA-sequencing (EGAS00001005161) [https://ega-archive.org/search-results.php?query=EGAS00001005161]. Gene set enrichment analyses used hallmark gene sets from MSIGDB v 5.2 [http://www.gsea-msigdb.org/gsea/msigdb/index.jsp]. Interactive visualizations of data can be explored in the Pediatric Brain Tumor Portal (pbtp.stjude.cloud). A reporting summary for this article is available as a Supplementary Information file. The main data supporting the findings of this study are available within the article and the Supplementary Figures, Tables and Data. The source data underlying Figs 8 and 9, and Supplementary Figs. 8b, 9a-c, 10a-d are provided in a supplementary Source Data file.

Field-specific reporting

Please select the one below that is the best fit for your research. If you are not sure, read the appropriate sections before making your selection.

- [x] Life sciences
- [ ] Behavioural & social sciences
- [ ] Ecological, evolutionary & environmental sciences

For a reference copy of the document with all sections, see nature.com/documents/nr-reporting-summary-flat.pdf

Life sciences study design

All studies must disclose on these points even when the disclosure is negative.

Sample size	Sample size for analyses with PDOX and cell line models used all available models and associated patient tumors and normal reference samples when available. Sample size of 5 or 6 for survival studies should be able to detect a magnitude of 2.5 std dev difference in the mean (and by extension with some regularity conditions in the median) with ~90% power and 5% type 1 error using a log rank test.
Data exclusions	No data excluded
Replication	All attempts at replication were successful. For genetic characterization of PDOX and cell line models, genome-wide sequencing was performed for all available models and matched patient tumor and germline DNA when available to show detection of signature mutations replicated in most cases, with some examples of subclonal populations changing between patient tumor and models. All drug screening was replicated by performing in triplicate. Additionally, as described in Results, after a large-scale screen of 1,134 drugs in nine cell lines., a collection of 93 drugs were selected as top hits and screened again in the same 9 cell lines and an additional 7 cell lines using an independently prepared compound plate to confirm that there were no errors in drug preparation or arrangement in screening plates and tested with a range of different doses. Western blots and immunohistochemistry were performed on a minimum of three samples per condition, as detailed in figure legends for each experiment and compared with vehicle controls as a reference.
Randomization	Mice for survival studies included mice over a baseline detectable level of bioluminescence randomly assigned among the 4 treatment groups using the RAND function in excel. For genome-wide molecular characterization of PDOX and cell line models, and drug screening of cell lines, all available models were analyzed and were not allocated into experimental groups.
Blinding	Dr. Chiang (neuropathology review) was blinded to the origin and genetic alterations of the PDOXs when evaluating the H&Es and when IHC and FISH were performed on PDOX samples. Blinding was not performed when directly comparing genome, transcriptome and methylome of PDOX and cell line models to their matched patient tumors, so blinding was not needed. Analysis of the molecular signatures of the entire cohort used unsupervised approaches. Blinding was not relevant for Western blotting where equal amounts of all samples were compared on the same blot and quantified.

Reporting for specific materials, systems and methods

We require information from authors about some types of materials, experimental systems and methods used in many studies. Here, indicate whether each material, system or method listed is relevant to your study. If you are not sure if a list item applies to your research, read the appropriate section before selecting a response.

Materials & experimental systems

n/a	Involved in the study	
[ ]	[x]	Antibodies
[ ]	[x]	Eukaryotic cell lines
[x]	[ ]	Palaeontology and archaeology
[ ]	[x]	Animals and other organisms
[ ]	[x]	Human research participants
[x]	[ ]	Clinical data
[x]	[ ]	Dual use research of concern

Methods

n/a	Involved in the study	
[x]	[ ]	ChIP-seq
[x]	[ ]	Flow cytometry
[x]	[ ]	MRI-based neuroimaging

Antibodies

Antibodies used	All antibodies and manufacturers are listed in Supplementary Table 6. The dilution used for each antibody is listed in Methods.
Validation	Akt Rabbit pAb (Cell Signaling, 9272) for Western control:<br>The validation is provided by Cell Signaling on the websites<br>https://www.cellsignal.com/datasheet.jsp?productId=9272&images=1

https://media.cellsignal.com/coa/9272/28/9272-lot-28-coa.pdf

Phospho-Akt (Ser473) Rabbit pAb (Cell Signaling, 9271) for Western and IHC
The validation is provided by Cell Signaling on the websites
https://www.cellsignal.com/datasheet.jsp?productId=9271&images=1
https://media.cellsignal.com/coa/9271/15/9271-lot-15-coa.pdf

p44/42 MAPK (Erk1/2) Rabbit pAb (Cell Signaling, 9102), for Western control.
The validation is provided by Cell Signaling on the websites
https://www.cellsignal.com/datasheet.jsp?productId=9102&images=0
https://media.cellsignal.com/coa/9102/27/9102-lot-27-coa.pdf

Phospho-p44/42 MAPK (Erk1/2) (Thr202/Tyr204) Rabbit pAb (Cell Signaling, 9101), for IHC and Western.
The validation is provided by Cell Signaling on the websites
https://www.cellsignal.com/datasheet.jsp?productId=9101&images=0
https://media.cellsignal.com/coa/9101/31/9101-lot-31-coa.pdf

Phospho-Histone H3 (Ser10) Rabbit pAb (Cell Signaling, 9701), for IHC.
The validation is provided by Cell Signaling on the websites
https://www.cellsignal.com/datasheet.jsp?productId=9701&images=1
https://media.cellsignal.com/coa/9701/17/9701-lot-17-coa.pdf

Cleaved Caspase-3 Rabbit mAb (BD, #559565), for IHC.
The validation is provided by BD Pharmingen™ on the website
https://www.bdbiosciences.com/ds/pm/tds/559565.pdf
Anti-ATRX rabbit pAb (Millipore Sigma, HPA001906), for IHC
The validation is provided by Millipore Sigma on the website
https://www.sigmaaldrich.com/catalog/product/sigma/hpa001906?lang=en&region=US

Anti-Nuclei Antibody, clone 235-1, mouse mAb (Millipore, MAB1281, Lot 2552340), for IF.
The validation is provided by Millipore Sigma on the website
https://www.sigmaaldrich.com/catalog/product/mm/mab1281?
lang=en&region=US&gclid=EAIaIQobChMI7eqQsLf47wIVxRrnCh38XQBMEAAYASAAEgI-e_D_BwE
in the supporting documents, Anti-Nuclei Antibody, clone 235-1 Certificates of Analysis

Anti-Mitochondria Antibody, surface of intact mitochondria, clone 113-1, mouse mAb (Millipore, MAB1273 Lot 2493926), for IF.
The validation is provided by Millipore Sigma on the website
https://www.sigmaaldrich.com/catalog/product/mm/mab1273?
lang=en&region=US&gclid=EAIaIQobChMIr6uI37f47wIVpCCtBh2HCwmgEAAYASAAEgJQLvD_BwE
in the supporting documents, Anti-Mitochondria Antibody, surface of intact mitochondria, clone 113-1 Certificates of Analysis
The anti-ATRX antibody was also validated in a CLIA-certified Anatomic Pathology Labortatory. The staining protocol for the ATRX antibody utilizing an automated instrument was first optimized using a tissue microarray of various normal tissue and then titrated using brain tumor tissue harboring a genetically proven pathogenic ATRX mutation. Validation was performed on tissue sections of 20 ATRX-mutant and 20 ATRX-wild type brain tumors and reviewed by two board-certified neuropathologists to ensure satisfactory performance. The validation process was repeated for each antibody lot. Positive and negative controls were included in each staining run and reviewed by a medical technologist experienced in antibody validation and then by a board-certified neuropathologist.

Eukaryotic cell lines

Policy information about cell lines

Cell line source(s)	Cell lines SJ-HGGX2c, SJ-HGGX6c, SJ-HGGX42c, SJ-HGGX39c, SJ-DIPGX7c, SJ-DIPGX9c, SJ-DIPGX29c, and SJ-DIPGX37c, were established in the Baker lab, SU-DIPG-IV, SU-DIPG-VI, SU-DIPG-XIII, SU-DIPG-XVII, SUDIPG-XIX, and SU-DIPG-XXI were obtained from Dr. Michelle Monje, and normal cell type references were human neural stem cells induced from H9 ES cells (Invitrogen, N7800-100), Human iPSC-derived astrocytes (Tempo Bioscience), and Human brainstem astrocytes (ScienCell Research Laboratories, #1840)..
Authentication	Cell lines were authenticated by STR profiling.
Mycoplasma contamination	Cell lines tested negative for mycoplasma contamination.
Commonly misidentified lines (See ICLAC register)	No commonly misidentified lines were used in this study.

Animals and other organisms

Policy information about studies involving animals; ARRIVE guidelines recommended for reporting animal research

Laboratory animals	Female CD1 nude mice were used for patient-derived orthotopic xenograft studies. Housing conditions for mice were temperature

	range 68 -74F, humidity range 30-70%, dark/light cycle cycle 12 hours dark/12 hours lightwith lights on at 6AM, off at 6PM.
Wild animals	Study did not involve wild animals
Field-collected samples	Study did not involve field-collected samples
Ethics oversight	St Jude Children's Research Hospital Animal Care and Use Committee approved animal experiments and Institutional Review Board approved use of human tissue and genome-wide sequencing.

Note that full information on the approval of the study protocol must also be provided in the manuscript.

Human research participants

Policy information about studies involving human research participants

Population characteristics	21 pediatric high-grade glioma samples were established as PDOX models and 19 were also subjected to genome-wide sequencing. The cohort consisted of 2 patients (9.5%) 2-5 years old, 11 (52%) 6-14 years old, 7 (33%) 15-19 years old, and one with age information not available. The cohort is 43% female and 57% male.
Recruitment	All available pediatric high-grade glioma samples were implanted and all models that successfully engrafted were included in this study.
Ethics oversight	Genomic analyses of patient material and use of patient tumor samples to establish xenografts and cell lines were performed with informed consent and approval from the St Jude Institutional Review Board of St. Jude Children's Research Hospital. Written informed consent was obtained from patients and/or legal guardians for use of tissue for research. This study complies with the Declaration of Helsinki and all other relevant ethical regulations.

Note that full information on the approval of the study protocol must also be provided in the manuscript.
